# The exotic species *Senecio inaequidens* pays the price for arriving late in temperate European grassland communities

**DOI:** 10.1007/s00442-019-04521-x

**Published:** 2019-10-01

**Authors:** Benjamin M. Delory, Emanuela W. A. Weidlich, Miriam Kunz, Joshua Neitzel, Vicky M. Temperton

**Affiliations:** 1 Ecosystem Functioning and Services, Institute of Ecology, Leuphana University, Universitätsallee 1, 21335 Lüneburg, Germany; 2 Present Address: Botanical Department, Universidade Federal de Santa Catarina, Florianópolis, Brazil

**Keywords:** Grassland invasion, Native community composition, Order and timing of arrival, Priority effects, Restoration

## Abstract

The exotic South African ragwort (*Senecio inaequidens* DC.) rapidly spread across Central Europe after its introduction, but we still do not know to what extent its timing of arrival in a plant community (i.e. before or after natives) and the composition of the native community being invaded affect (1) its capacity to invade a European grassland, (2) the performance of the native species, and (3) the direction and strength of priority effects. In a greenhouse experiment, we manipulated the timing of arrival of the exotic species (*Senecio*) and the composition of the native community to test the influence of these factors on the productivity and N content of exotic and native species. We also investigated if the plant species origin (native or exotic) and the native community composition affected the benefit of arriving early and the cost of arriving late in the community. The establishment success of *Senecio* strongly depended on its timing of arrival in a grassland community. *Senecio* benefited more from arriving early than did the natives. The presence of legumes in the community did not favour invasion by *Senecio*. When natives arrived later than *Senecio*, however, priority effects were weaker when legumes were part of the native community. Our results showed that inhibitory priority effects created by natives can lower the risk of invasion by *Senecio*. An early arrival of this species at a site with low native species abundance is a scenario that could favour invasion.

**Electronic supplementary material:**

The online version of this article (10.1007/s00442-019-04521-x) contains supplementary material, which is available to authorized users.

## Introduction

Invasion of plant communities by non-native (“exotic” or “alien”) species is largely recognized as one of the main drivers of biodiversity loss worldwide (Sala et al. 2000; Elbakidze et al. 2018). In Europe, the number of alien vascular plant species has increased steadily since the beginning of the nineteenth century, mainly because of enhanced economic activities such as trade and tourism increasing the risk of invasion (Elbakidze et al. 2018). Amongst all the exotic invasive plant species introduced to Europe, the South African ragwort (*Senecio inaequidens* DC., Asteraceae; hereafter referred to as *Senecio* throughout the article) is often considered as a fast spreading invader (Lachmuth et al. 2010) and this is probably linked to its ability to colonize a wide range of ecological habitats (Heger and Böhmer 2005). *Senecio* is a large perennial forb whose seeds have been repeatedly introduced to locations in Central Europe via the transport of sheep wool imported from the Eastern highlands of South Africa (Ernst 1998; Lachmuth et al. 2010). It was observed for the first time in Europe (West Germany) at the end of the nineteenth century (Ernst 1998; Heger and Böhmer 2005) and, after a time lag of nearly 80 years, it started to spread across Germany, mainly from a population introduced in Belgium at the beginning of the twentieth century (Lachmuth et al. 2010). It is an early-successional ruderal species requiring open sites with little resource competition and is mainly found on disturbed sites, including along railway tracks (Heger and Böhmer 2005).

The invasion success of an exotic species in a new environment is multifactorial (Seastedt and Pyšek 2011). Most of the research in this area has focused on two complementary aspects of plant invasion. A first area of research deals with the identification of traits favouring the invasiveness of exotic species, such as high competitive ability (Sakai et al. 2001; Perkins and Hatfield 2014), high propagule pressure and phenotypic plasticity (Kolar and Lodge 2001; Allendorf and Lundquist 2003; Wainwright and Cleland 2013), the ability to reproduce vegetatively/clonal growth (Kolar and Lodge 2001), or the ability to produce allelochemicals that suppress local species at new sites (Callaway and Aschehoug 2000; Callaway and Ridenour 2004; Aschehoug et al. 2014). A second area of research deals with the identification of native plant community characteristics affecting their susceptibility to invasion (invasibility), such as the disturbance regime (Chytrý et al. 2008; Seastedt and Pyšek 2011), resource availability (Davis et al. 2000; Zefferman et al. 2015; Liu et al. 2018), species and functional group richness (Tilman 1997; Knops et al. 1999; Naeem et al. 2000; Wardle 2001; Kennedy et al. 2002; Fargione and Tilman 2005; Pokorny et al. 2005; Scherber et al. 2010; Mason et al. 2017), species and functional group composition (Crawley et al. 1999; Prieur-Richard et al. 2002; Fargione et al. 2004; Wardle et al. 2011; Byun et al. 2013; Yannelli et al. 2017), and the presence of natural enemies (Keane and Crawley 2002; Shea and Chesson 2002; Levine et al. 2004). Because exotic species often germinate more quickly, grow faster, and take up resources more efficiently than native species (Wainwright et al. 2012; Wilsey et al. 2015), the invasion process is also tightly linked to the concept of priority effect in ecology, in which the species arriving first at a site significantly affect the development, growth, and reproduction of species arriving later (Chase 2003; Vannette and Fukami 2014; Temperton et al. 2016).

In grasslands, priority effects caused by biotic interactions can have effects that supersede abiotic influence on the community. Priority effects caused by species arriving before others can affect community structure as well as ecosystem functioning both aboveground (Wilsey et al. 2015; Weidlich et al. 2017) and belowground (Körner et al. 2008; Weidlich et al. 2018). Such priority effects occur either because the early-arriving species reduce the amount of resources (water, nutrients, space, etc.) available for late-arriving species (called niche pre-emption) (Fukami 2015), or because the early-arriving species modified the type of niches available for the species arriving later via, for instance, extra nitrogen (N) availability if N_2_-fixing species arrive first, root exudation or the selection of a particular soil microbiome (called niche modification, including plant–soil feedbacks) (Callaway et al. 2004; Suding et al. 2013; van der Putten et al. 2013; Perkins and Hatfield 2014; Fukami 2015). Despite its importance for the restoration of sites threatened by exotic species invasion, we still know very little about how differences in timing of arrival between exotic and native species affect native–exotic relationships in European grasslands (but see Lang et al. 2017). Depending on how the development, growth, or reproduction of late-arriving species is affected by species that arrive early during assembly, priority effects can be either positive (i.e. late species favoured by early species) or negative (i.e. late species inhibited by early species). During ecological restoration of degraded landscapes, there is much potential for creating negative priority effects by sowing natives before the arrival of exotics (Hess et al. 2019). If exotic species with strong competitive abilities are given a head-start (e.g. after a disturbance), however, they can quickly outcompete or exclude the establishment of native species (Abraham et al. 2009; Grman and Suding 2010; Stevens and Fehmi 2011; Dickson et al. 2012; Wainwright et al. 2012; Ulrich and Perkins 2014; Wilsey et al. 2015; Stuble and Souza 2016), and such priority effects can persist for several years (Martin and Wilsey 2012; Vaughn and Young 2015).

To what extent the timing of arrival of *Senecio* (i.e. before or after natives) affects its capacity to invade a European grassland community is not yet known but, considering that (1) it is an early-colonizing species able to produce a large amount of wind-dispersed seeds (Ernst 1998), (2) it has the capacity to colonize and survive in mature grassland communities (Scherber et al. 2003), and (3) introduced *Senecio* populations respond to greater resource availability by increasing aboveground and belowground productivity and reproductive output (Bossdorf et al. 2008), it is likely that an early arrival of this species (i.e. before natives) could lead to successful invasion of grassland communities. In contrast, we expect that negative priority effects created by sowing natives before the arrival of the exotic species would lower the risk of invasion of grassland communities by *Senecio*.

Both direct and indirect facilitation between invading exotic species (Simberloff and Von Holle 1999; Flory and Bauer 2014) or between natives and exotics (Bruno et al. 2003; Bulleri et al. 2008; Saccone et al. 2010) are important invasion mechanisms. When looking at the effect of natives on exotics, facilitation in the form of N fertilization by leguminous species as well as nurse plant interactions have been shown to increase communities’ susceptibility to invasion (Maron and Connors 1996; Mason et al. 2013). With regard to grassland ecosystems, the presence of legumes is known to increase the amount of N available for its neighbours via two co-occurring mechanisms: (1) direct N transfer (N transfer) from legumes to non-legume neighbours, and (2) reduced interspecific competition for soil mineral N (N sparing) when legumes derive most of their N from the atmosphere (Temperton et al. 2007). How such facilitation mechanisms affect the invasiveness of an exotic species has been the central issue in many research studies, with some showing that the presence of N_2_-fixing species in the community can favour invasion (Prieur-Richard et al. 2000, 2002; Mwangi et al. 2007; Scherber et al. 2010), and others not showing such positive relationship between invasibility and legume presence (Tilman 1997). In contrast, the presence of strong competitors, such as grasses, tends to increase the resistance of a plant community to invasion (Prieur-Richard et al. 2000, 2002; Fargione et al. 2004; Mwangi et al. 2007; Scherber et al. 2010). As described above, both the species and functional group composition of a native plant community can have important consequences for invasion but, surprisingly, little is known about the effect of this factor on the establishment of *Senecio* in European grasslands. Because N facilitation can be expected when legumes are present in a community, we hypothesize that grassland communities containing N_2_-fixing species would be more susceptible to invasion by *Senecio* than communities containing only grasses. In other words, we expect to find weaker priority effects of native species on *Senecio* if legumes are present in the community being invaded (i.e. *Senecio* benefits more than natives from the presence of legumes that arrived earlier than it did). Similarly, when the exotic species is the first to arrive in the community, we also expect to find weaker priority effects acting on the late-arriving native species if legumes are present (i.e. greater functional complementarity reducing interspecific competition).

In this paper, we present the results of a controlled greenhouse experiment designed to evaluate the benefits of arriving early and the costs of arriving late (i.e. priority effects) for the exotic species *Senecio* colonizing a community composed of species characteristic of mesotrophic temperate European grasslands. The costs and benefits for native species arriving earlier or later than *Senecio* were also evaluated in this study. In addition to time of arrival, we also manipulated the composition of the native community to test if this would affect the strength and direction of priority effects for both native and exotic species. Here, we addressed the following questions:

1. How does the invasiveness of *Senecio* in a European grassland community depend on its timing of arrival and the composition of the native community?
2. How is the performance of the native species affected by the timing of arrival of *Senecio* and the composition of the native community?
3. Does *Senecio* benefit more than the native species from arriving early in the community?
4. Does the composition of the native plant community affect the direction and strength of priority effects for exotic and native species?
5. Do the native species pay a greater cost than *Senecio* when arriving late in the community?

## Materials and methods

### Plant material

All seeds used for the experiment described in this paper were native wild species, not cultivars, provided by Rieger-Hofmann GmbH (Blaufelden-Raboldshausen, Germany).

### Experimental design

**Fig. 1:**
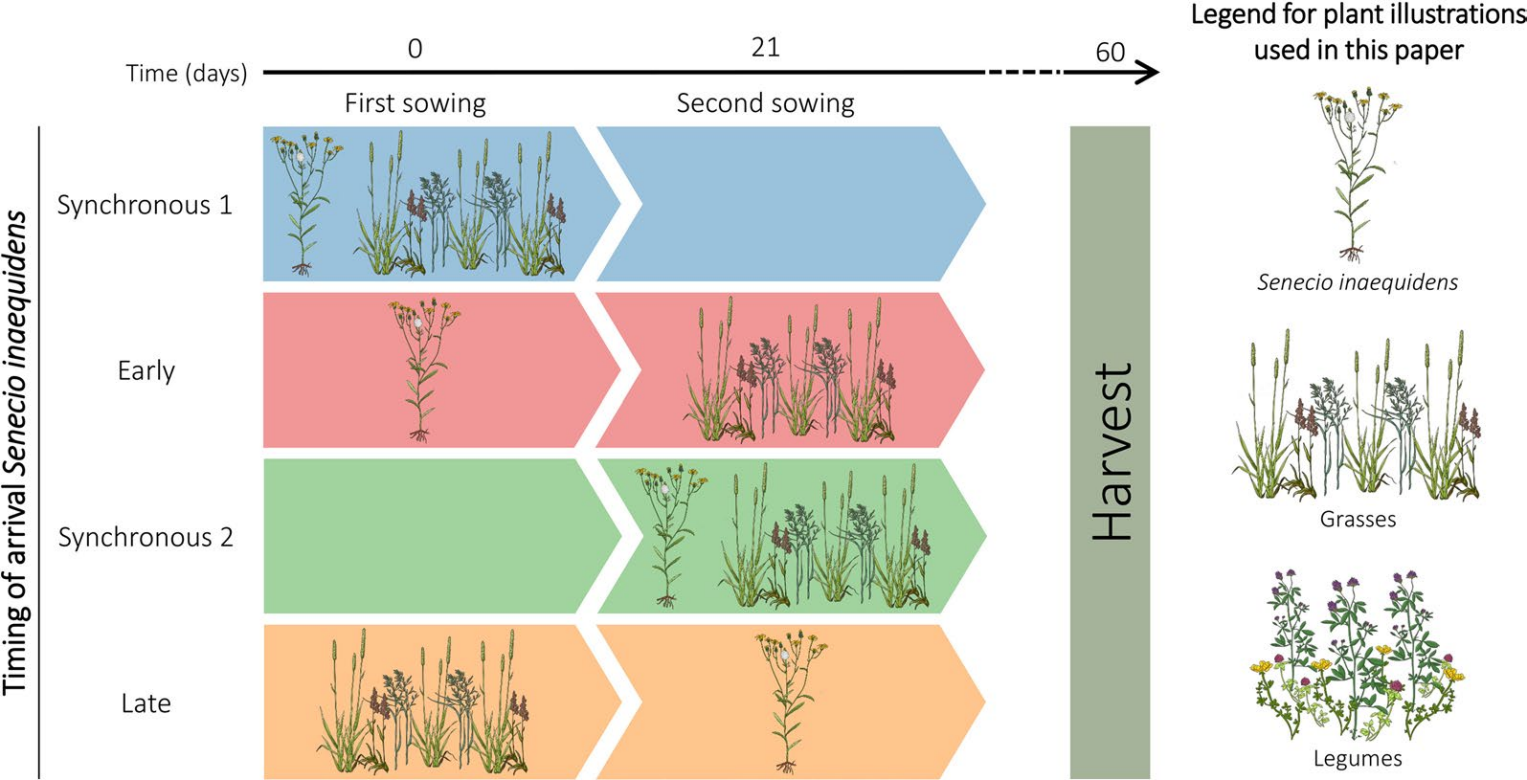
Illustration of one scenario for manipulating arrival of *Senecio* relative to the native community that was tested in this study. A second scenario in which *Senecio* was sown a community composed of grasses and legumes was also tested (not shown). Note that, as shown in this figure and all subsequent figures, the terms “late” or “early” always refer to the timing of arrival of the exotic species, not the background community. The colour code is the same as in Fig. 6. A legend for plant illustrations used throughout this paper is provided on the right side of the figure. Plant illustrations by Carolina Levicek. The colour version of the figure is available online

We set up an experiment using a full factorial and randomized design to test the influence of two main factors on the performance of an exotic (*Senecio inaequidens*) and native species characteristic of temperate mesotrophic European grasslands: (1) the timing of arrival of the exotic species in the plant community, and (2) the composition of the native plant community. With regard to the timing of arrival of the exotic species, it was sown either earlier, later, or at the same time (synchronous) as the native species. Because manipulating the timing of arrival of the exotic species implied the organization of two sowing events, two synchronous treatments were set up so that all priority effect treatments could be directly compared to plants that had grown for the same length of time: one at the first sowing (Synchronous 1), and another one at the second sowing (Synchronous 2). Therefore, the factor timing of arrival of the exotic species comprised a total of four levels (Fig. 1). Two different native plant communities were used in this experiment. In the first scenario, *Senecio* was sown into a community composed of grasses only (*Holcus lanatus*, *Festuca pratensis*, *Phleum pratense*). In the second scenario, it was sown into a community composed of grasses (*H. lanatus*, *F. pratensis*, *P. pratense*) and legumes (*Medicago sativa*, *Trifolium pratense*, *Lotus corniculatus*). Each of the 8 treatment combinations (4 timing of arrival of *Senecio* × 2 native plant community compositions) was replicated 5 times. This experiment was set up in a greenhouse located in Rotes Feld, Lüneburg (Lower Saxony, Germany). During the period of the experiment, the temperature inside the greenhouse was 23.5 ± 5.2 °C during the day and 16.8 ± 2.8 °C during the night.

Eight days before the start of the experiment, 5 L of sand was added at the bottom of 40 pots (volume: 18.4 L, top surface area: 28 × 28 cm^2^, height: 30 cm). Then, the pots were filled with a mixture of sand (50%, v/v) and potting soil (50%, v/v) up to the top. After filling, the pots were placed inside a greenhouse and were watered twice before the start of the experiment. We started the experiment by sowing the species arriving first in the communities (Fig. 1). Depending on the timing of arrival of the exotic species, we sowed either *Senecio* (Early arrival), the native species (Late arrival), or both *Senecio* and the native species (Synchronous 1). A grassland experiment that tested the influence of priority effects as well as sowing density and interval (comparing three and 6 weeks) on aboveground productivity found that although sowing interval played a role, the priority effect was much larger (von Gillhaussen et al. 2014). Based on this as well as many priority effect studies using a three-week sowing interval (Ulrich and Perkins 2014; Wilsey et al. 2015), we decided to sow the rest of the species in the pots of our experiment 21 days after the first sowing (Fig. 1). During this second sowing event, we sowed either the native species (Early arrival), *Senecio* (Late arrival), or both *Senecio* and the native species (Synchronous 2). After each sowing event, a thin layer of sand and potting soil (50%/50%, v/v) was added at the top of the pots to favour germination. For each treatment, the sowing density of each species was adjusted to take into account differences in plant composition and germination rates so to allow for an even community outcome. When the native community was composed of grasses only, the sowing density was adjusted to reach a target of 50 individuals/species growing in the pots. When the native community was composed of a mixture of grasses and legumes, however, the sowing density was adjusted to reach a target of 25 individuals/species. For all treatments, the sowing density of the exotic species was calculated to reach a target of 25 individuals growing in each pot. The composition and sowing densities used for each plant community are summarized in Online Resource 1. All pots were regularly watered using tap water throughout the duration of the experiment.

Sixty days after the first sowing event, the aboveground biomass was harvested separately for *Senecio*, the legumes, and the grasses. Plants were healthy and did not show obvious signs of pathogen or herbivore attack. Shoot samples were then dried at 70 °C until constant mass was reached.

Since all plant individuals used in this experiment were grown under the same experimental conditions, the differences in biomass production between Synchronous 1 and Synchronous 2 plants are mainly due to the difference in growth duration between the two treatments (i.e. Synchronous 1 plants grew for a total of 60 days, while Synchronous 2 plants grew for a total of 39 days). Temperature variations during the experiment are unlikely to explain the differences in biomass production observed between synchronous treatments, as the average day and night temperatures for the first 21 days (day: 24.9 °C; night: 18.6 °C) and the last 39 days of growth (day: 24.0 °C; night: 18.7 °C) were very similar.

### Measurements

For each pot, we measured the total shoot dry weight of *Senecio*, legumes, and grasses. The total carbon (%C) and nitrogen (%N) content in *Senecio,* legume, and grass shoots (the latter were pooled into grass or legume biomass) was determined with a C/N analyser (Vario EL; Elementar, Langenselbold, Germany) using 14.7 ± 1.5 mg of dry and finely ground material. Evidence for N facilitation in communities containing legumes was investigated using the δ^15^N natural abundance method (sensu Temperton et al. 2007). Sample δ^15^N values (‰) were calculated using Eq. 1, where R represents the ratio of ^15^N/^14^N isotopes. *R*_*sample*_ values were determined using an elemental analyser (Elementar Vario EL Cube) coupled to a stable isotope ratio mass spectrometer (IR-MS, Isoprime). Isotope ratios were determined for *Senecio*, grasses, and legumes using dried and finely ground plant material (*Senecio*: 7.6 ± 1.1 mg; grasses: 6.7 ± 0.1 mg; legumes: 9.0 ± 0.1 mg). Atmospheric N_2_ is the international standard used for IR-MS measurements of δ^15^N (*R*_standard_). If legumes were actively fixing atmospheric N_2_, *R*_sample_ values measured in their shoots should be very close to the *R*_standard_ value measured in the atmosphere. Therefore, the expected δ^15^N value measured in the shoots of actively fixing legumes should be close to zero. For species relying solely on soil N, however, we expect δ^15^N values to be different from zero (in theory, close to the soil δ^15^N value) because soil N often has a greater ^15^N abundance than atmospheric N_2_ (Unkovich et al. 2008). Apparent N transfer between legumes and non-legume neighbours was assessed by comparing the δ^15^N values measured in *Senecio* and grass shoots growing with or without legumes in the community. If belowground N transfer occurred, we expect non-legume shoots to have lower δ^15^N values when growing in communities containing leguminous species (Temperton et al. 2007). Because non N_2_-fixing species can also benefit from the soil N that is not taken up by leguminous species (N sparing), we used both the N status (%N) and the δ^15^N values measured in plant shoots to investigate how N dynamics were affected by the timing of arrival of the exotic species and the species composition of the native community.

1 $$\delta^{15} {\text{N}} = \left( {\frac{{R_{\text{sample}} }}{{R_{\text{standard}} }} - 1} \right) \times 1000.$$

### Quantification of priority effects

In assembly research, the strength of priority effects has mainly been quantified using interaction indices in the form of log response ratios (Vannette and Fukami 2014; Stuble and Souza 2016). Because such indices are not bounded between finite values, they are however not well suited for comparing results between different experiments (Díaz-Sierra et al. 2017). Here, we introduce a set of standardized, symmetric, and bounded interaction indices designed to quantify the benefit of arriving early (*B*) and the cost of arriving late (*P*) during community assembly (Table 1; note that in our definition of priority effects, these only occur as effects on later arriving species). These indices share the same mathematical properties as the relative interaction index commonly used to measure competition and facilitation between interacting plants, i.e. they are standardized, symmetric around zero, and are bounded between − 1 and + 1 (Díaz-Sierra et al. 2017). To the best of our knowledge, this study is the first to use a set of interaction indices derived from the well-known relative interaction index to quantify the benefit of arriving early and the cost of arriving late during assembly. The direction of the priority effect is given by the sign of *P*, with inhibitory priority effects having negative values, and facilitative priority effects having positive values. The strength of the priority effect is given by the absolute value of *P*. Because the calculation of *B* and *P* relies on the comparison of the performance of organisms arriving at different time in the community, but having the same age at harvest (e.g. *Senecio* growing in Early and Synchronous 1 treatments; see Fig. 1), they can only be calculated if the experiment includes as many synchronous treatments (i.e. all species arriving at the same time) as sowing events. The values for *P* and *B* reported in this paper were all calculated using shoot dry weight data. The total biomass of native species in the community was calculated by summing the biomass of legumes and grasses. In the supplementary material, cost and benefit values calculated using shoot N content data are also provided (Online Resource 5).

**Table 1:** Quantifying the benefit of arriving early and the cost of arriving late during assembly. In the equations, *Y* is a particular response variable (e.g. biomass production, N content, etc.)

	For the exotic species	For the native species	Interpretation		
			Negative values	0	Positive values
Benefit (B) of arriving early	$B_{E} = \frac{{Y_{E}^{Early} - Y_{E}^{Sync1} }}{{Y_{E}^{Early} + Y_{E}^{Sync1} }}$	$B_{N} = \frac{{Y_{N}^{Late} - Y_{N}^{Sync1} }}{{Y_{N}^{Late} + Y_{N}^{Sync1} }}$	Negative effect of early arrival	No benefit	Positive effect of early arrival
Cost (P) of arriving late	$P_{E} = \frac{{Y_{E}^{Late} - Y_{E}^{Sync2} }}{{Y_{E}^{Late} + Y_{E}^{Sync2} }}$	$P_{N} = \frac{{Y_{N}^{Early} - Y_{N}^{Sync2} }}{{Y_{N}^{Early} + Y_{N}^{Sync2} }}$	Negative (or inhibitory) priority effect	No priority effect	Positive (or facilitative) priority effect

The superscripts and subscripts refer to the timing of arrival of the exotic species (as shown in Fig. 1) and the origin of the plant species on which *Y* was measured (*E* is for exotic, *N* is for natives), respectively

### Statistical analyses

Generalised linear models (GLMs) were used to investigate the effect of the timing of arrival of the exotic species (Arrival), the species composition of the native plant community (Composition), and their interaction (Interaction) on the aboveground biomass productivity of the exotic species and the grasses. Because the number of *Senecio* individuals that established varied from pots to pots, we tested if adding the number of *Senecio* individuals that were actually growing in the pots as a covariate would improve the quality of the models. After comparing models fitted with and without the number of *Senecio* individuals, we did not find any evidence to support that models accounting for the number of *Senecio* individuals were better than the models that did not account for it. In addition, there was no correlation between the number of *Senecio* individuals growing in a pot and the total productivity achieved by the exotic species (*r* = 0.15, *P* = 0.35). Therefore, in this paper, we only report the results of statistical models fitted without using the number of *Senecio* individuals growing in the pots as a covariate. When the interaction term did not significantly improve the model, a new model without interaction term was fitted. GLMs were also used to investigate the effect of the timing of arrival of the exotic species on the aboveground productivity of the legumes. GLMs were always fitted on plant biomass data using a Gamma distribution and a log-link function.

The effect of the timing of arrival of the exotic species, the species composition of the native plant community, and their interaction on the C/N content and δ^15^N values measured in *Senecio* and grass shoots was investigated using two-way ANOVA models. One-way ANOVA models were used to test for the effect of the timing of arrival of the exotic species on the C/N content and δ^15^N values measured in legume shoots.

Two-way ANOVA models were used to test if the plant species origin (native or exotic), the native community composition, and their interaction had an effect on the benefit of arriving early (*B*_*E*_ or *B*_*N*_) and the cost of arriving late (*P*_*E*_ or *P*_*N*_) in the community. When reported, 95% confidence intervals were computed by bootstrapping (1000 iterations) using the percentile method. We considered that the mean value of a group was not significantly different from zero when the 95% confidence interval of that group included zero.

General linear hypotheses (post hoc tests) were tested using Tukey contrasts and the glht function of the multcomp R package (Hothorn et al. 2008). When results of post hoc tests are shown, adjusted P values (single-step method) are reported to account for multiple comparisons of group means. All statistical analyses were performed in R 3.4.3 (R Core Team 2018) with an alpha value of 0.05.

## Results

### Timing of arrival and native plant community composition affect the performance of *Senecio*

**Fig. 2:**
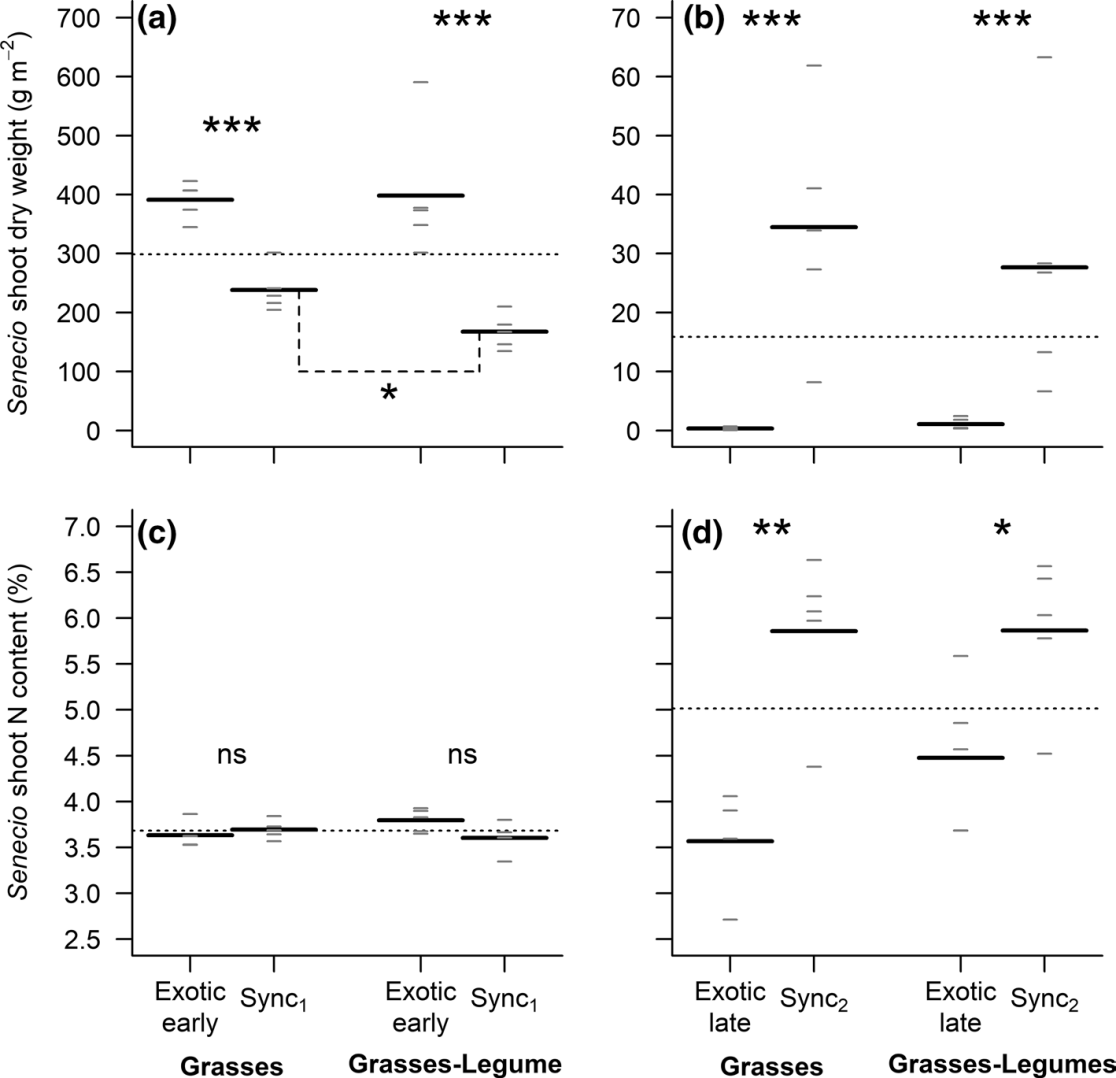
*Senecio*’s shoot dry weight and shoot N content under **a**, **c** early and **b**, **d** late timing of *Senecio* arrival in two native communities differing in species composition. All panels show the overall mean (horizontal dotted line), the mean of each group (long black horizontal lines, *n* = 4–5), and each individual observation (short grey horizontal lines). Note that the terms “late” or “early” in all graphs always refer to the timing of arrival of the exotic species (Fig. 1). Because of the low shoot dry weight of *Senecio* obtained for one replicate of the treatment where the exotic species arrived late in a community made of native grass species only, we were not able to measure the N content of that replicate, leaving *n* = 4 in this case. ns, not significant (*P *> 0.05); *P < 0.05; **P < 0.01; ***P < 0.001

When *Senecio* was the first species sown in a community, it produced significantly more biomass than when sown at the same time as the native species (Fig. 2a). The effect of the timing of arrival of *Senecio* on plant productivity, however, varied with the species composition of the native community (Interaction: *F*_1,16_ = 5.31, *P* = 0.03). If the native plant community contained both grasses and legumes, the biomass gain due to an early sowing of *Senecio* (+ 138%) was larger than when the native community contained only grasses (+ 64%). This result can be explained by the fact that, when both exotic and native species were sown at the same time, *Senecio* was less productive in communities containing legumes than in communities containing only grasses (Fig. 2a; *z* = 2.96, *P* = 0.01), while the biomass achieved by *Senecio* did not differ between both communities when it was sown earlier than the native species (Fig. 2a; *z* = − 0.15, *P* = 1.0). Whatever the composition of the native plant community, an early arrival of *Senecio* did not affect its N content (Fig. 2c; Arrival: *F*_1,16_ = 1.19, *P* = 0.29; Composition: *F*_1,16_ = 0.37, *P* = 0.55; Interaction: *F*_1,16_ = 4.33, *P* = 0.05). With regard to the C content of *Senecio* shoots, it was negatively affected by an early arrival of *Senecio* (− 3.8%; Arrival: *F*_1,16_ = 9.29, *P* = 0.01; Composition: *F*_1,16_ = 1.13, *P* = 0.30; Interaction: *F*_1,16_ = 1.88, *P* = 0.19), particularly when the native community was composed of legumes and grasses (Online Resource 2a).

When the exotic species was sown later than the natives, it barely managed to establish and produced between 96% and 99% less biomass than the plants that were sown at the same time as the native species (Fig. 2b; Arrival: *F*_1,16_ = 62.74, *P* < 0.001). When sown later than the natives, the biomass achieved by *Senecio* did not depend on the composition of the native community (Composition: *F*_1,16_ = 1.16, *P* = 0.30; Interaction: *F*_1,16_ = 3.19, *P* = 0.09). For both plant community compositions, a late arrival of *Senecio* had a strong negative effect on its shoot N content (Fig. 2d; arrival: *F*_1,15_ = 24.90, *P* < 0.001). Although we did not find any significant effect of the species composition of the native plant community on the N content of *Senecio* (Fig. 2d; Composition: *F*_1,15_ = 0.83, *P* = 0.38; Interaction: *F*_1,15_ = 1.53, *P* = 0.23), the decrease in N content associated with a late arrival of the exotic species was lower in communities containing legumes (− 24%) than in communities containing only grasses (− 39%). For both native community compositions, the C content of *Senecio* shoots was similar whether it was sown later or at the same time as the native species and did not differ between the two plant communities (Online Resource 2b; Arrival: *F*_1,15_ = 10^−4^, *P* = 1.0; Composition: *F*_1,15_ = 0.94, *P* = 0.35; Interaction: *F*_1,15_ = 1.74, *P* = 0.21).

### Timing of arrival of the exotic species and plant community composition affect the biomass production and N content of native grass species

**Fig. 3:**
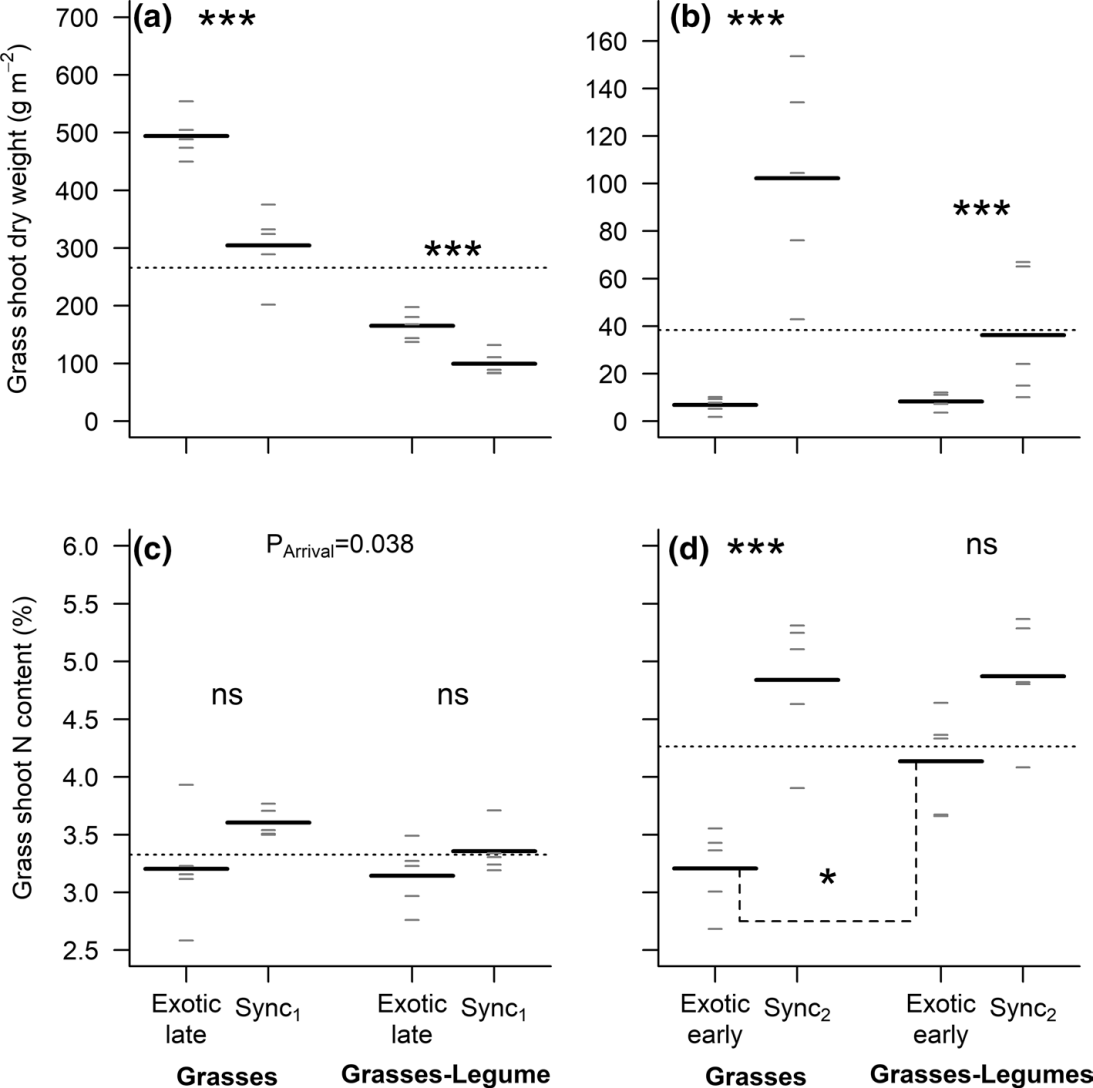
Grasses’ shoot dry weight and shoot N content under **a**, **c** late and **b**, **d** early timing of *Senecio* arrival in two native communities differing in species composition. All panels show the overall mean (horizontal dotted line), the mean of each group (long black horizontal lines, *n* = 5), and each individual observation (short grey horizontal lines). Note that the terms “late” or “early” in all graphs always refer to the timing of arrival of the exotic species, such that the “late” treatment outcome shows the performance of grasses when they arrive early (Fig. 1). ns, not significant (*P *> 0.05); *P < 0.05; **P < 0.01; ***P < 0.001

In comparison with a situation where both native and exotic species were sown at the same time, the aboveground biomass of the grass species was between 62% and 66% greater if they were sown earlier than the exotic (Fig. 3a; Arrival: *F*_1,16_ = 41.61, *P* < 0.001), and this result was independent of plant community composition (Interaction: *F*_1,16_ = 0.02, *P* = 0.88). When the natives were sown before the exotic species, the timing of arrival of the exotic species was the only factor affecting the shoot N content (Fig. 3c; Arrival: *F*_1,16_ = 5.12, *P* = 0.04; Composition: *F*_1,16_ = 1.27, *P* = 0.28; Interaction: *F*_1,16_ = 0.48, *P* = 0.50). On average, the grasses had a lower N content when they were sown earlier than the exotic species. This difference in N content was no longer significant when the effect of the timing of arrival of the exotic species was investigated separately for each plant community (Fig. 3c).

Overall, the grasses established poorly when they were sown later than the exotic species. In this situation, they produced between 77% and 93% less biomass than the grasses that were sown at the same time as *Senecio* (Fig. 3b). This timing of arrival effect, however, was dependent on the native community composition (Interaction: *F*_1,16_ = 4.84, *P* = 0.04), mainly because the lower grass density in communities containing legumes led to a lower grass biomass production in these communities when all species arrived at the same time (Fig. 3b). On average, the grasses sown later than *Senecio* also had a significantly lower N content (− 24%) than the grasses that were sown at the same time as the exotic species, particularly when native communities did not contain any legumes (Fig. 3d; Arrival: *F*_1,16_ = 30.18, *P* < 0.001; Composition: *F*_1,16_ = 4.96, *P* = 0.04; Interaction: *F*_1,16_ = 4.31, *P* = 0.05). For both native community compositions, the N content of grass shoots was lower when the native species were sown after the exotic, but the difference with the synchronous treatment was only significant when legumes were absent (Fig. 3d; *t* = 5.35, *P* < 0.001). In addition, when *Senecio* was the first species to arrive, the N content in grass shoots was 29% greater if the native community included legumes (Fig. 3d; *t* = − 3.04, *P* = 0.03).

Neither timing of arrival of the exotic species nor the species composition of the native community affected the C content of grass tissues (Online Resource 3).

### Timing of arrival of the exotic species affects the biomass production and N content of native legume species

**Fig. 4:**
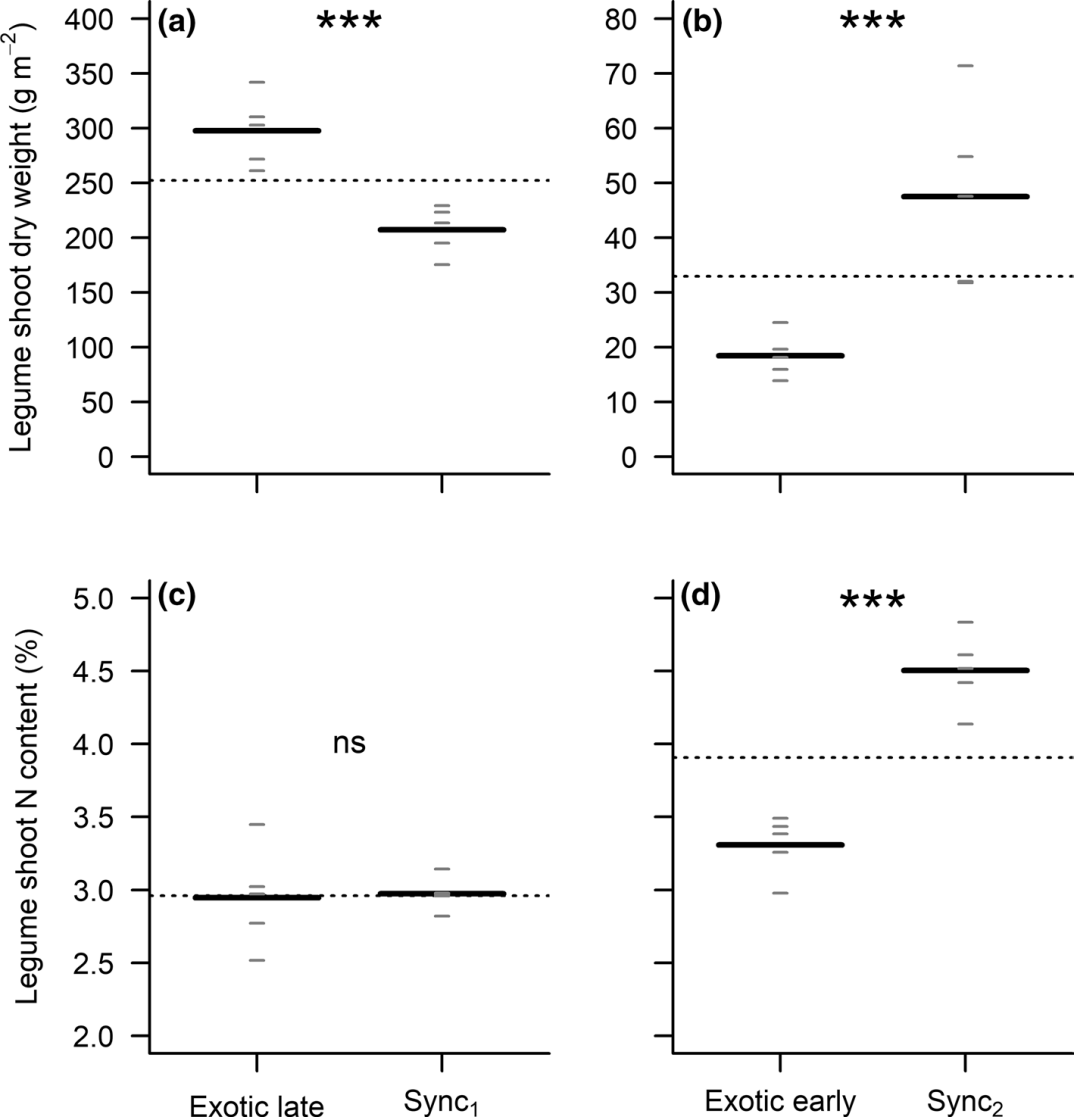
Legumes’ shoot dry weight and shoot N content under **a**, **c** late and **b**, **d** early timing of *Senecio* arrival. All panels show the overall mean (horizontal dotted line), the mean of each group (long black horizontal lines, *n* = 5), and each individual observation (short grey horizontal lines). Note that the terms “late” or “early” in all graphs always refer to the timing of arrival of the exotic species, such that the “late” treatment outcome shows the performance of legumes when they arrive early (Fig. 1). ns, not significant (*P *> 0.05); *P < 0.05; **P < 0.01; ***P < 0.001

When legumes were sown before *Senecio*, they were 44% more productive than when sown at the same time as the exotic species (Fig. 4a; *F*_1,8_ = 27.87, *P* < 0.001). We did not observe any effect of a late arrival of the exotic species on the N content (Fig. 4c; *F*_1,8_ = 0.03, *P* = 0.87) and C content (Online Resource 4a; *F*_1,8_ = 0.88, *P* = 0.38) of legume shoots.

When *Senecio* was the first species to be sown in the community, the aboveground biomass and the shoot N content of the legumes decreased by 61% (Fig. 4b; *F*_1,8_ = 25.54, *P* < 0.001) and 27% (Fig. 4d; *F*_1,8_ = 66.36, *P* < 0.001) in comparison with control plants sown at the same time as the exotic species, respectively. An early arrival of the exotic species did not have an effect on the C content of legume tissues (Online Resource 4b; *F*_1,8_ = 0.01, *P* = 0.91).

### *Senecio* benefits more from arriving early than the natives

**Fig. 5:**
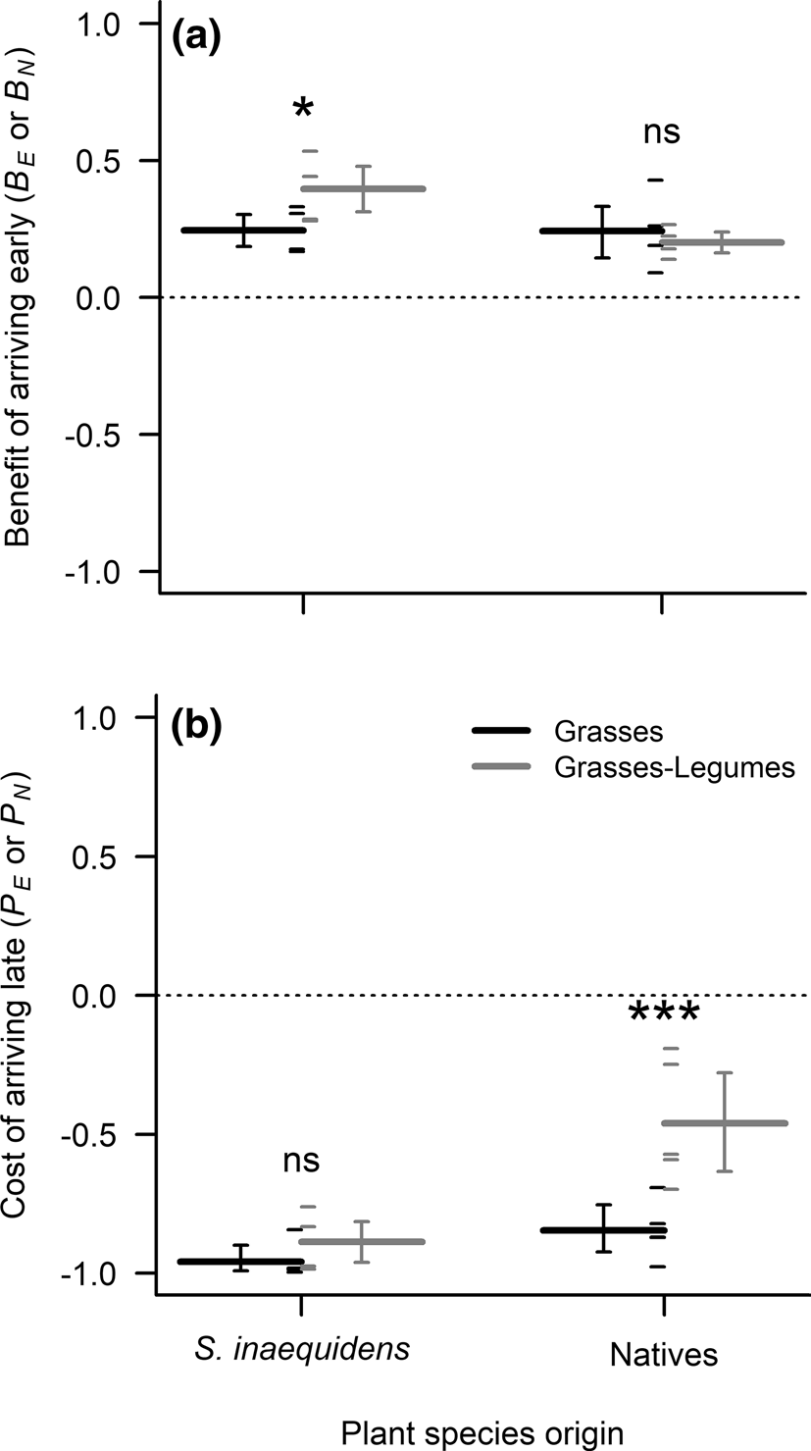
**a** Benefit of arriving early and **b** cost of arriving late in the community for exotic and native species. Benefits and costs were calculated based on shoot dry weight data using the equations listed in Table 1. All panels show the mean of each group (long horizontal lines, *n* = 5), and each individual observation (short horizontal lines). Results are shown separately for each plant species origin (exotic or natives) and each native community composition. Error bars are 95% confidence intervals computed by bootstrapping using the percentile method. If a 95% confidence interval does not include zero, the mean value of the group is significantly different from zero. ns, not significant (*P *> 0.05); *P < 0.05; **P < 0.01; ***P < 0.001

Our results showed that both exotic and native species benefited from arriving early in the community but, on average, *Senecio* benefited more than the natives (Fig. 5a; Species: *F*_1,16_ = 5.56, *P* = 0.03). Interestingly, the effect of the native community composition on the benefit of arriving early differed between exotic and native species (Fig. 5a; Interaction: *F*_1,16_ = 5.31, *P* = 0.03). The positive effect associated with an early arrival was greater for the exotic species if it was followed by a mixture of grasses and legumes compared with a mixture of grass species only (*t* = 2.56, *P* = 0.04). For the natives, however, the benefit of arriving early was similar for both community compositions (*t* = -0.70, *P* = 0.74). Very similar results were obtained when the benefit of arriving early was calculated using shoot N content data (Online Resource 5).

### Arriving late is less costly for natives than *Senecio*, particularly when legumes are present in the community

Both exotic and native species created inhibitory priority effects for species arriving later (Fig. 5b). Overall, growth reduction by priority effects was strongest for the exotic species (Species: *F*_1,16_ = 19.45, *P* < 0.001) and when legumes were absent from the plant community (Community: *F*_1,16_ = 13.97, *P* = 0.002). In addition, the composition of the native community affected differently the priority effect strength measured on exotic and native species arriving later (Interaction: *F*_1,16_ = 6.63, *P* = 0.02). Our results showed that the strength of priority effects acting on *Senecio* did not depend on the composition of the native community it tried to invade (*t* = 0.82, *P* = 0.66). The strength of priority effects acting on native species, however, was significantly affected by the composition of the native community. In comparison with a scenario where the native community is composed of grasses only, inhibitory priority effects acting on natives following an early establishment of the exotic species were on average 46% weaker when legumes were present in the native community (*t* = 4.46, *P* < 0.001). Very similar results were obtained when the strength of priority effects was calculated using shoot N content data (Online Resource 5).

### Evidence for atmospheric N_2_ fixation by legumes, but not for N transfer to non-legume neighbours

**Fig. 6:**
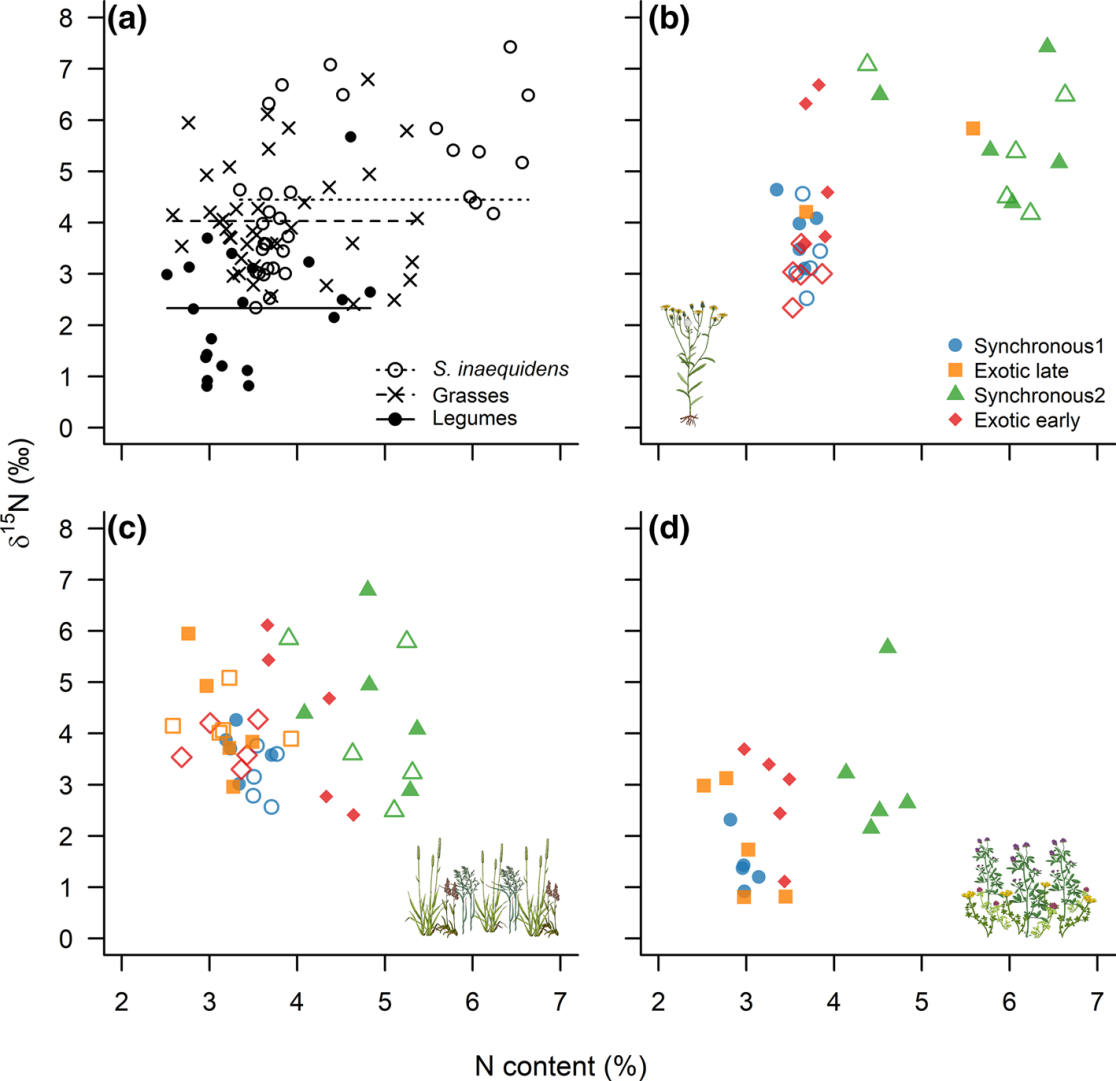
Relationship between the N content and the δ^15^N values measured in **a**, **b***Senecio*, **a**, **c** grass, and **a**, **d** legume shoots. **a** Shows all data together. The horizontal lines represent the mean δ^15^N values calculated for *Senecio*, the grasses, and the legumes (see legend **a**). **b**–**d** Dots are coloured based on the timing of arrival of the exotic species (see key in **b**). Open symbols refer to native communities containing only grasses, while closed symbols refer to native communities containing grasses and legumes. Because of the low shoot dry weight of *Senecio* obtained for one replicate of the treatment where the exotic species arrived late in a community made of native grass species only, we were not able to measure the N content of that replicate, giving *n* = 4 for this treatment. For the same reason, we were not able to measure the δ^15^N values in all replicates of the treatment where *Senecio* arrived late in a community composed of grass species only, as well as in three replicates of the treatment where *Senecio* arrived late in a community composed of grasses and legumes. The colour version of the figure is available online

On average, the δ^15^N values measured in legume shoots were 48% and 42% lower than those measured in *Senecio* and grass shoots, respectively (Fig. 6a). This result strongly suggests that the legumes harvested at the end of the experiment were actively fixing atmospheric N_2_. However, the positive δ^15^N values measured in legume shoots also indicate that the legumes did not rely solely on the fixation of atmospheric N_2_ as a source of N for plant growth, but also on the soil N pool.

The δ^15^N values measured in the exotic species were affected by its timing of arrival (Arrival: *F*_2,24_ = 11.54, *P* < 0.001) and by the composition of the native community being invaded (Composition: *F*_1,24_ = 6.19, *P* = 0.02), but not by the interaction between these two factors (Interaction: *F*_2,24_ = 2.11, *P* = 0.14) (Fig. 6b). Surprisingly, *Senecio* shoots had greater δ^15^N values when legumes were present in the community (*t* = 3.10, *P* = 0.005). In addition, *Senecio* shoots from the Synchronous 2 treatment also had greater δ^15^N values than the ones harvested from the Early (*t* = 3.94, *P* = 0.002) and Synchronous 1 treatments (*t* = 3.41, *P* = 0.006).

When looking at the native species, the δ^15^N values measured in grass shoots were affected neither by the timing of arrival of the exotic species (Arrival: F_3,32_ = 1.59, *P* = 0.21) nor by the species composition of the native plant community (Composition: F_1,32_ = 1.17, *P* = 0.29; Interaction: F_3,32_ = 0.11, *P* = 0.95) (Fig. 6c). Similarly, there was only weak support that the timing of arrival of the exotic species affected the δ^15^N values of legume shoots (Arrival: F_3,16_ = 2.86, *P* = 0.07) (Fig. 6d).

Despite that legumes were actively fixing atmospheric N_2_ during our experiment, the fact that the shoot δ^15^N values measured in *Senecio* and native grass species were not lower when grown in communities containing legumes did not support direct N transfer from legume to non-legume neighbours.

## Discussion

The results presented here confirmed that the timing of arrival of *Senecio* has a strong impact on its performance. This exotic species performed much better (greater biomass production) when it was the first species to arrive in the community. In fact, pots in which the exotic species was sown before the natives were near monocultures, with *Senecio* accounting for 96% of the total aboveground biomass, while it accounted for only 40% of the total biomass when it was sown at the same time as the native species. On the other hand, it barely managed to establish (less than 0.6% of the total aboveground biomass) and had a lower N content when it arrived later. The timing of arrival of the exotic species in the community also strongly affected the performance of the native species. When the natives arrived 3 weeks before *Senecio*, they produced more biomass than natives arriving at the same time as the exotic species. When the natives arrived later in the community, however, they established poorly (lower biomass) and had a lower shoot N content. Therefore, whatever the origin of the plant species arriving first (either exotic or native), it created strong inhibitory priority effects for the species arriving later in the assembly process. These results are in line with previous studies that have shown that priority effects with either positive (lower abundance of exotic species) or negative (natives outcompeted by exotics) conservation outcomes can be created depending on whether the exotic species arrive later or earlier than the natives, respectively (Abraham et al. 2009; Grman and Suding 2010; Stevens and Fehmi 2011; Dickson et al. 2012; Wainwright et al. 2012; Ulrich and Perkins 2014; Wilsey et al. 2015; Stuble and Souza 2016).

Using nectar-inhabiting microorganisms as a model system, Vannette & Fukami (2014) demonstrated that the priority effects are stronger if (1) species arriving at different time in the community use resources in a similar way (niche overlap), (2) early-arriving species have a strong impact on the local environment (impact niche), and (3) the growth, survival, and reproduction of late-arriving species is greatly affected by environmental conditions (requirement niche). Because both exotic and native species created strong inhibitory priority effects in our experiment, it is likely that the species that were sown first impacted the environment in such a way that it degraded the requirement component of the late-arriving species’ niches. In an attempt to classify the mechanisms behind the creation of priority effects during community assembly, Fukami (2015) distinguished two main categories of mechanisms by which early-arriving species impact their local environment and affect the establishment of species arriving later. The first mechanism, referred to as niche pre-emption, is mainly resource driven. It is based on the assumption that the plant species arriving first at a site affects species arriving later by reducing the amount of available resources such as light, water, soil nutrients, and physical space itself. The second mechanism, referred to as niche modification, is based on the assumption that the species arriving first in the community will modify the types of niches available locally and will affect the identity of the species that will be able to colonize the community. Contrary to niche pre-emption mechanisms, which can only lead to the creation of inhibitory priority effects, niche modification mechanisms can lead to the creation of both facilitative (e.g. soil fertilization by leguminous species) or inhibitory priority effects (e.g. exudation of allelochemicals altering the growth rate of species arriving later) (Maron and Connors 1996; Callaway and Aschehoug 2000; Fukami 2015). Which class of mechanisms led to the creation of priority effects in our experiment is not clear yet, but the near competitive exclusion and lower N content of species arriving late in the community strongly suggest that niche pre-emption mechanisms played an important role (Fukami 2015). In addition, we did not find any evidence of apparent N transfer from legumes to non-legume neighbours in our experiment, thus not supporting niche modification by leguminous species via N fertilization.

*Senecio* being an early-successional species (Ernst 1998; Heger and Böhmer 2005), it is likely to be one of the first species to arrive at open sites, particularly in disturbed and stony areas. Our results showed that *Senecio* benefited more from arriving early in the community than the native species, and this effect was particularly strong when the following mixture included legumes. This result is in agreement with the hypothesis that, due to their earlier emergence, greater germination rates, and faster growth, exotics would benefit more than native species from arriving early in the community (Dickson et al. 2012; Wainwright et al. 2012; Wilsey et al. 2015; Hess et al. 2019). This result, however, contradicts other priority effect studies that showed that exotic species benefited equally (Stuble and Souza 2016) or less (Cleland et al. 2015) than natives when they were the first to arrive in a community. As suggested by Stuble & Souza (2016), differences between studies could arise from differences between species and study systems (e.g. testing annuals vs perennials).

Overall, *Senecio* suffered more from arriving late than the native species. This key result contradicts previous studies that showed that the cost of arriving late in a plant community tends to be lower for exotics than for natives (Stuble and Souza 2016), or that exotics create stronger priority effects than natives (Wilsey et al. 2015). Arriving late was less costly for the native species than for the exotic species, suggesting a possible evolutionary adaptation of native grassland species to finding free niches despite high canopy cover of the community into which they are trying to establish. Interestingly, our results also showed that the strength of the priority effects acting on the exotic species did not depend on the composition of the native community being invaded. Contrary to our expectations, the growth inhibition of *Senecio* when it arrived in a community composed of native grasses only was as strong as when it arrived in a mixture of grasses and legumes. Because (1) the native grass community had a greater N content per unit plant biomass than the community composed of a mixture of grasses and legumes (Online Resource 6), and (2) the two native communities did not differ in productivity across our priority effect treatments (Online Resource 7), the grass community seemed better at taking up soil N than the grass–legume mixture in our experiment.

Even though the amount of soil N available for plant growth was probably greater in the community containing legumes, the fact that the strength of the priority effects acting on *Senecio* was not different between the two native communities used in our experiment strongly suggests that available soil N was not the limiting factor for the establishment of the exotic species in our artificial grassland communities. Instead, pre-emption of light or other soil resources by natives might be more important mechanisms to explain the near competitive exclusion of late-arriving *Senecio* invaders (Ernst 1998; Heger and Böhmer 2005; Frankow-Lindberg 2012; Wilsey et al. 2015). The composition of the native plant community, however, had a strong impact on the priority effects created by the exotic species on late-arriving natives. When a mixture of native grasses and legumes followed *Senecio*, these priority effects were nearly 50% weaker than when legumes were absent from the native community. Although we found evidence for N sparing in communities containing legumes, as shown by the greater N content of late-arriving *Senecio* or grass species when legumes were present (Fig. 6), we cannot fully conclude that the decrease in priority effect strength observed for the grass–legume mixture was solely due to N facilitation associated with the presence of N_2_-fixing species in the community, mainly because the two native community compositions used in this study differed in species and functional group richness.

There is now an expanding body of the literature claiming that the creation of priority effects would be a useful technique to restore degraded habitats, alter competitive relationships, and steer plant communities towards desirable states in terms of biodiversity and functioning (Wilsey et al. 2015; Temperton et al. 2016; Weidlich et al. 2017, 2018; Young et al. 2017). Manipulating plant community assembly to promote native species that will ultimately exert strong priority effects on exotics is also a very interesting approach to lower the risk of invasion (Hess et al. 2019). To the best of our knowledge, this study is one of the few that explicitly tested how historical contingency by priority effects impact on the establishment of a rapidly expanding exotic species in European grasslands (Lang et al. 2017). Priority effects being contingent on environmental conditions during plant establishment (Young et al. 2017), their effects on biotic interactions and community structure and functioning are particularly hard to predict, thus presenting a major challenge for plant ecologists. For priority effects to be useful in invasive species management, further research is needed. First, a better understanding of the mechanisms behind the creation of such priority effects is essential for improving the predictive power of ecology. For instance, although niche pre-emption by early-arriving species played a role in our study, we cannot exclude the possibility that other niche modification mechanisms, such as the production of allelochemicals by early-arriving species or plant–soil feedbacks, have occurred. Second, because environmental severity affects the strength of facilitative interactions (Brooker et al. 2007), priority effects (Vannette and Fukami 2014; Young et al. 2017) and invasive success in general (Zefferman et al. 2015), additional experiments are needed to determine how the timing of arrival of *Senecio* in native grassland communities and facilitative interactions with natives (either direct or indirect) affect invasion across an environmental stress gradient (e.g. disturbance, resource availability). Finally, we argue that long-term experiments are needed to elucidate how weather conditions during plant establishment affect the strength, direction, and persistence of priority effects (Temperton et al. 2016).

Altogether, our results have implications for managing the risk of invasion of European grasslands by *Senecio inaequidens*. The poor establishment of *Senecio* that we observed when it arrived only three weeks after natives suggests that dense grassland communities are unlikely to be invaded. If a disturbance leading to a significant reduction in native species abundance occurs, however, an early arrival of *Senecio* is a scenario that could potentially favour invasion. *Senecio* has indeed many characteristics favouring its invasiveness. Each individual is able to produce a large amount of seeds (between 10,000 and 29,000 achenes) that will be dispersed by wind or animals from short to great distances, thus imposing a high propagule pressure on the local environment (Ernst 1998; López-García and Maillet 2005). These seeds then accumulate in the soil where they can persist for several years, particularly if they are buried and not directly located at the soil surface (López-García and Maillet 2005). Both their high degree of dormancy polymorphism and their capacity to resist frost (-15 °C) contribute to their persistence in the soil seed bank (Ernst 1998). Under favourable conditions, seeds produced by *Senecio* can germinate quickly and seedlings are known to have high relative growth rates, particularly under non-limiting nitrogen supply (Ernst 1998; López-García and Maillet 2005). As disturbed habitats are usually associated with increased nutrient availability and lower competition pressure from natives, it is probable that all the biological characteristics described above will favour invasion by increasing differences in competitive ability between natives and *Senecio*. This is especially true if *Senecio* was already present in or next to the disturbed area because seeds are then likely to be present in the soil seed bank. In this situation, increasing native propagule pressure by sowing fast germinating species would be a very interesting strategy to lower the risk of invasion, although the strength of the priority effect will probably depend on the time difference between the germination of native and exotic species. Overall, minimizing the creation of open spaces and niches for *Senecio* to arrive early is important to lower the risk of invasion, which could be better achieved in mown rather than grazed grasslands.

## Electronic supplementary material

Below is the link to the electronic supplementary material.

Supplementary material 1 (PDF 534 kb)

## Data Availability

Raw data and R scripts used for data analysis are fully accessible here: 10.5281/zenodo.2558204
