# Supplementary material for: The exotic species Senecio inaequidens pays the price for arriving late in temperate European grassland communities
Source: Oecologia. 2019 Oct 1;191(3):657–71. doi: 10.1007/s00442-019-04521-x (PMC6825039; doi:10.1007/s00442-019-04521-x)
Supplement: Supplementary file 1 — Supplementary material 1 (PDF 534 kb) [file 442_2019_4521_MOESM1_ESM.pdf]

Supplementary information

The exotic species *Senecio inaequidens* pays the price for arriving late in temperate European grassland communities

Benjamin M. Delory[1,*], Emanuela W.A. Weidlich[1,2], Miriam Kunz[1], Joshua Neitzel[1], Vicky M. Temperton[1,*]

[1] Ecosystem Functioning and Services, Institute of Ecology, Leuphana University, Universitätsallee 1, 21335 Lüneburg, Germany

[2] Current address: Botanical Department, Universidade Federal de Santa Catarina, Florianópolis, Brazil

[*] Corresponding authors: Benjamin M. Delory and Vicky M. Temperton

**Authors**	**Contact information**	**ORCID**
Benjamin M. Delory	Benjamin.Delory@leuphana.de	0000-0002-1190-8060
Emanuela W.A. Weidlich	emanuela.ww@gmail.com	0000-0002-2098-6140
Vicky M. Temperton	Vicky.Temperton@leuphana.de	0000-0003-0543-4521

Online Resource 1. Species composition and sowing densities used in our experiment for each plant community. Plant illustrations by Carolina Levicek

**Species**	**Plant illustration**	**Germination rate (%)**	**Number of seeds/pot**	
			Native grasses only	***Native grasses and legumes***
Trifolium pratense (native)	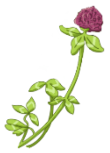	60	0	42
Lotus corniculatus (native)	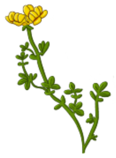	60	0	42
Medicago sativa (native)	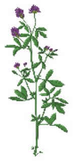	35	0	71
Holcus lanatus (native)	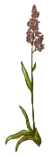	95	53	26
Phleum pratense (native)	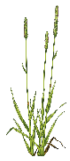	82.5	61	30
Festuca pratensis (native)	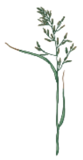	65	77	38
Senecio inaequidens (exotic)	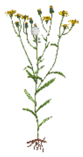	70	36	36

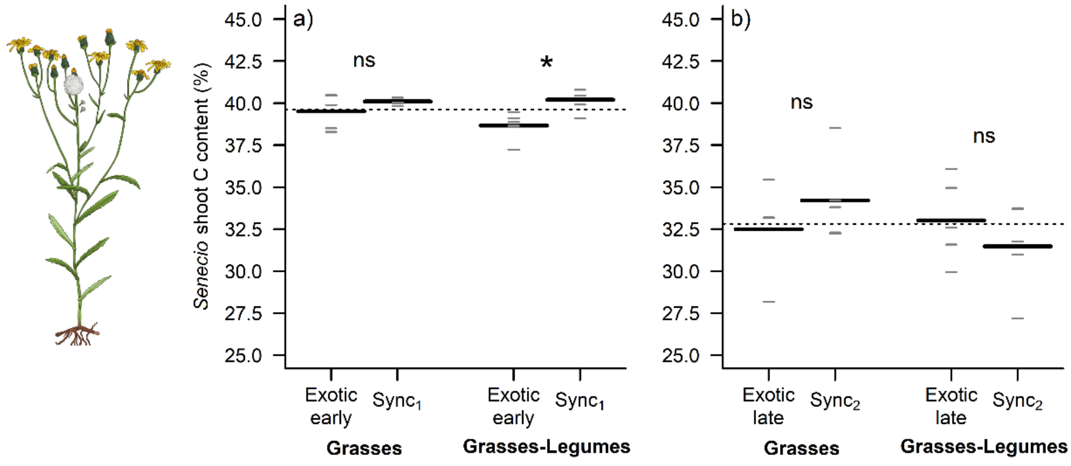

Online Resource 2. *Senecio*'s shoot C content under a) early and b) late timing of *Senecio* arrival in two native communities differing in species composition. Each graph shows the overall mean (horizontal dotted line), the mean of each group (horizontal black lines, n=4-5), and each individual observation (short horizontal grey lines). Please note that the terms "late" or "early" in all graph always refer to the timing of arrival of *S. inaequidens* (Fig. 1). Because of the low shoot dry weight of *S. inaequidens* obtained for one replicate of the treatment where the exotic species arrived late in a community made of native grass species only, we were not able to measure the C content of that replicate. ns, not significant (P>0.05); *, P<0.05; **, P<0.01; ***, P<0.001

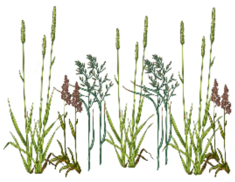

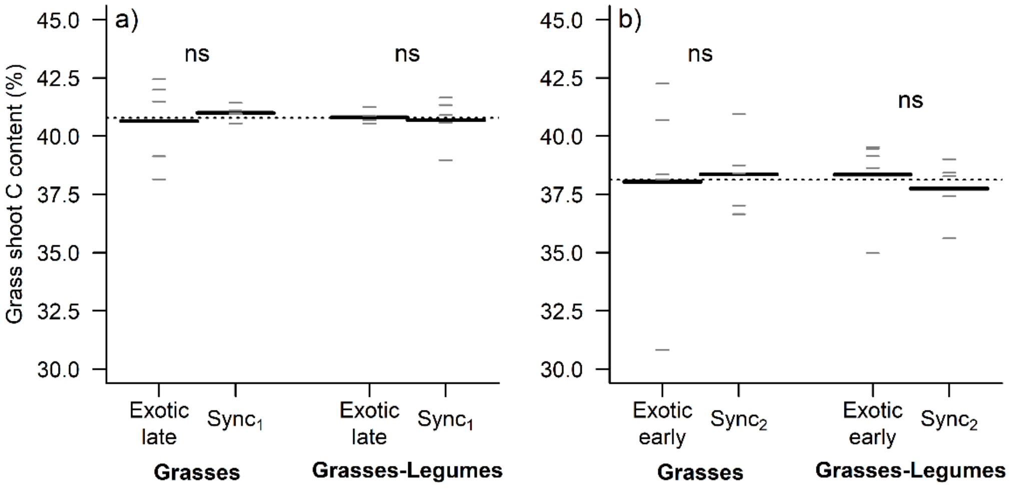

Online Resource 3. Grasses' shoot C content under a) late and b) early timing of *Senecio* arrival in two native communities differing in species composition. Each graph shows the overall mean (horizontal dotted line), the mean of each group (horizontal black lines, n=5), and each individual observation (short horizontal grey lines). Please note that the terms "late" or "early" in all graphs always refer to the timing of arrival of *S. inaequidens* (Fig. 1). ns, not significant (P>0.05); *, P<0.05; **, P<0.01; ***, P<0.001

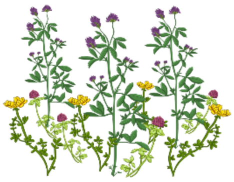

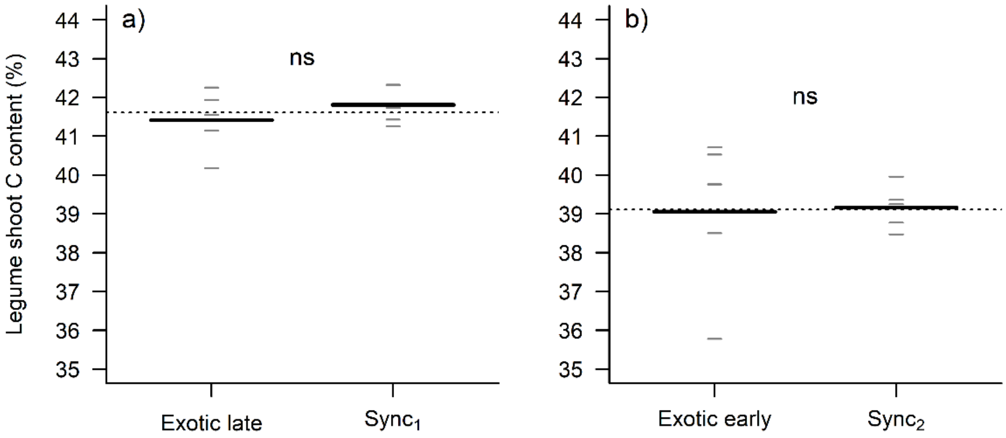

Online Resource 4. Legumes' shoot C content under a) late and b) early timing of *Senecio* arrival. Each graph shows the overall mean (horizontal dotted line), the mean of each group (horizontal black lines, n=5), and each individual observation (short horizontal grey lines). Please note that the terms "late" or "early" in all graphs always refer to the timing of arrival of *S. inaequidens* (Fig. 1). ns, not significant (P>0.05); *, P<0.05; **, P<0.01; ***, P<0.001

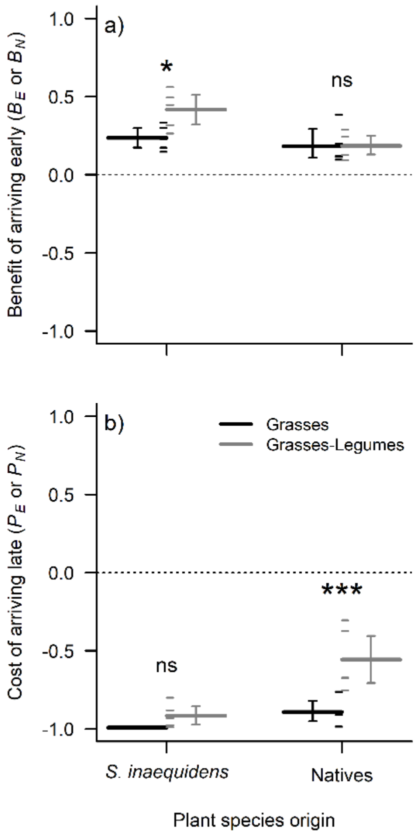

Online Resource 5. a) Benefit of arriving early and b) cost of arriving late in the community for exotic and native species. Benefits and costs were calculated based on shoot N content data using the equations listed in Table 1. All panels show the mean of each group (long horizontal lines, n=4-5), and each individual observation (short horizontal lines). Because of the low shoot dry weight of *S. inaequidens* obtained for one replicate of the treatment where the exotic species arrived late in a community made of native grass species only, we were not able to measure the N content of that replicate. Results are shown separately for each plant species origin (exotic *S. inaequidens* or natives) and each native community composition (see key). Error bars are 95% confidence intervals computed by bootstrapping using the percentile method. If a 95% confidence interval does not include zero, the mean value of the group is significantly different from zero. ns, not significant (P>0.05); *, P<0.05; **, P<0.01; ***, P<0.001

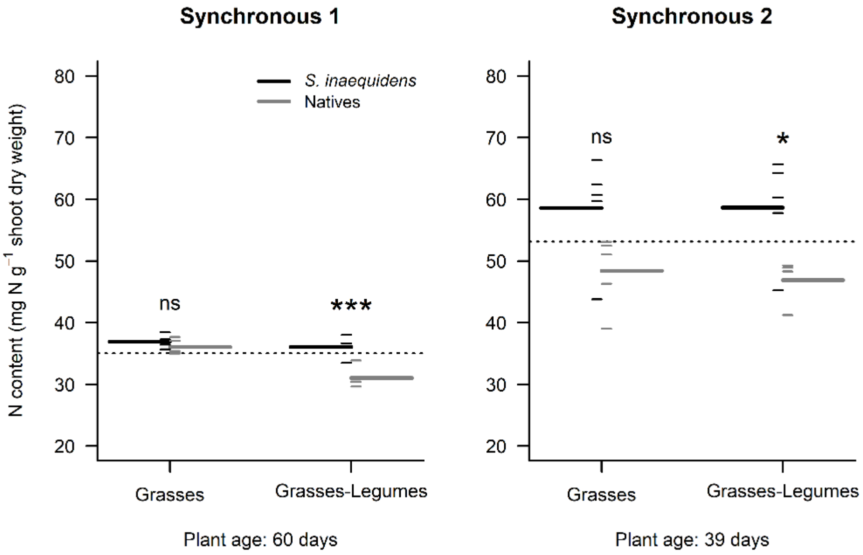

Online Resource 6. Shoot N content of exotic and native species in plant communities in which all species arrived at the same time. All panels show the overall mean (horizontal dotted line), the mean of each group (long horizontal lines, n=5), and each individual observation (short horizontal lines). Results are shown separately for each native community composition (horizontal axis) and each species origin (exotic *S. inaequidens* or natives, see key). We used the shoot N concentration in the exotic species and the native communities as a proxy for their ability to take up nitrogen from the environment. The results presented here show that *S. inaequidens* took up as much N per unit biomass as a community composed of three native grasses only. When the native community was a mixture of grasses and legumes, however, the N concentration in shoot tissues was on average lower than that measured in the exotic species, thus suggesting a lower ability of the grass-legume mixture to take up nitrogen. The same trends were observed in Synchronous 1 and Synchronous 2 communities. ns, not significant (P>0.05); *, P<0.05; **, P<0.01; ***, P<0.001

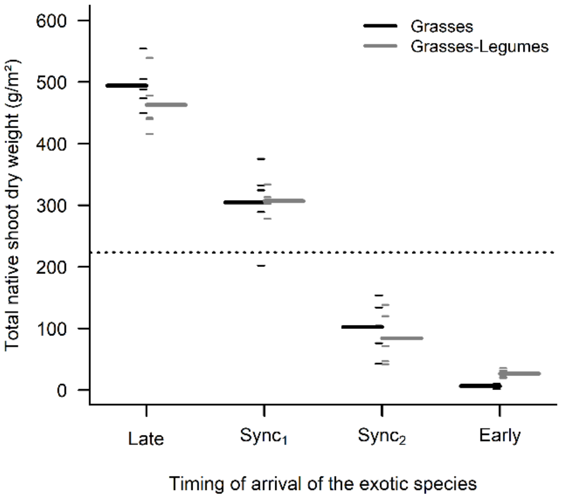

Online Resource 7. The productivity of the native plant communities did not depend on their species composition. The figure shows the overall mean (horizontal dotted line), the mean of each group (long horizontal lines, n=5), and each individual observation (short horizontal lines). Results are shown separately for each priority effect treatment (horizontal axis) and each native community composition (see key)
